# Childhood psychopathology dimensions as predictors of mood disorders among offspring at high risk of mood and substance use disorders

**DOI:** 10.1192/j.eurpsy.2025.658

**Published:** 2025-08-26

**Authors:** C. L. Vandeleur, S. Ranjbar, M.-P. F. Strippoli, E. Castelao, P. Marquet, M. Preisig

**Affiliations:** 1 Psychiatry, Lausanne University Hospital, Lausanne, Switzerland

## Abstract

**Introduction:**

Prospective longitudinal research on the offspring of parents with mood and substance use disorders allows to identify specific symptoms that occur before the onset of full-blown mood episodes in these offspring at high-risk of mood disorders.

**Objectives:**

The goal of the present analysis was to assess early psychopathology dimensions measured by the Child Behavior Checklist (CBCL), completed by parents and separately by their offspring, as potential clinical manifestations occurring before the onset of mania/hypomania or major depressive disorder (MDD) in these offspring.

**Methods:**

As part of a family study, we have collected information on 105 interviewed probands with bipolar disorder, 76 with MDD, 21 with substance use disorders (SUD) and 101 controls, as well as on their 239 children with follow-up information. The mean age of the offspring at study intake was 10.0 (s.d. 4.4) years and the mean follow-up duration was 16.3 (s.d: 5.6) years. The CBCL was completed by the parents describing their offspring as well as by the offspring themselves at their first assessment. Parent-offspring correlation coefficients were calculated and associations of the CBCL dimensions with the subsequent risk of mania/hypomania or MDD in offspring according to either informant were established using multinomial logistic regression models, adjusted for demographics, parental mood disorders or SUD and intrafamilial correlations.

**Results:**

According to offspring reports, 17 of them developed mania/hypomania and 92 MDD over the follow-up. Offspring reported more withdrawn/depressed symptoms as predictors of mania/hypomania than controls, whereas parents reported their offspring to have more anxious/depressed symptoms before mania/hypomania. As predictors of MDD, offspring reported more withdrawn/depressed symptomatology but less somatic complaints than controls, whereas no significant predictors of MDD were identified by parents. Among the dyads where both offspring and parents had provided information (n=206), offspring were less likely to report rule-breaking behaviors as predictors of mania/hypomania, whereas their parents reported the opposite association. Parent-offspring correlations for all CBCL dimensions were statistically significant, ranging from 0.16 to 0.32, the correlation being 0.21 for rule-breaking behaviors.

**Conclusions:**

Offspring and parental reports of predictors of mood disorders were discordant and the parent-child agreement for CBCL dimensions in the restricted sample was moderate. Whereas parent-offspring concordance for depressive symptoms as predictors of mania/hypomania in offspring was higher than for anxious or somatic problems, the reports of rule-breaking behaviors as predictors showed the highest discordance. This underscores the necessity to include information from multiple informants in the assessment of predictors of mood disorders in offspring.

**Disclosure of Interest:**

None Declared

